# Silent Spring, the 50^th^ anniversary of Rachel Carson’s book

**DOI:** 10.1186/1472-6785-12-20

**Published:** 2012-09-27

**Authors:** David Pimentel

**Affiliations:** 1 College of Agriculture and Life Sciences, Cornell University, Ithaca, NY, 14853, USA

## Abstract

**Editorial:**

David Pimentel is a professor of ecology and agricultural sciences at Cornell University, Ithaca, NY 14853–0901. His Ph.D. is from Cornell University and had postdoctoral research at the University of Chicago, MIT, and fellowship at Oxford University (England). He was awarded a distinguished honorary degree from the University of Massachusetts. His research spans the fields of energy, population ecology, biological pest control, pesticides, sustainable agriculture, land and water conservation, livestock, and environmental policy. Pimentel has published more than 700 scientific papers and 37 books and has served on many national and government committees including the National Academy of Sciences; President’s Science Advisory Council; U.S Department of Agriculture; U.S. Department of Energy; U.S. Department of Health, Education and Welfare; Office of Technology Assessment of the U.S. Congress; and the U.S. State Department. He is currently Editorial Advisor for *BMC Ecology*. In this article, he reflects on 50 years since the publication of Rachel Carson’s influential book, Silent Spring.

## What was Silent Spring about?

Rachel Carson’s book “Silent Spring” [1] was published 50 years ago. This is one of the most influential books of all time because of its information on the effects of widespread pesticide use on the environment. The book had a tremendous impact on environmental protection, food production, and human health, and raised the vital issue of the toxicity of pesticides on human health, bird populations, fisheries (including salmon), and numerous other animal species. I was only a graduate student when Rachel Carson’s publications hit the ecological and pest control literature. The impact of her publications in the United States and worldwide were tremendous!

## So who was Rachel Carson?

Carson was a broadly trained biologist with the U.S. Bureau of Fisheries. In her work she was concerned for human health, fishes, birds, insects, plants, and all organisms in the environment. She is credited with the start of applied ecology throughout the world.

As she worked for the U.S. Bureau of Fisheries, she was fully aware of the decline in young salmon populations that was occurring in Northwest Miramichi River after the pesticide DDT (**d**ichloro**d**iphenyl**t**richloroethane) was first sprayed for control of the spruce budworm. She pointed out that the pesticide spray killed spruce budworm pest as well as the aquatic insects that the young salmon depended on for food. Thus, with the food supply for the young salmon destroyed, their populations declined.

## What sparked her into writing Silent Spring?

Pesticide poisonings were a serious problem in public health and environment even in their early use. In the U.S., a dramatic impact developed from spraying DDT in the 1950’s to protect elm trees from the Dutch Elm Disease spread by a small bark beetle. Heavy spraying for the bark beetle control resulted in indirectly killing robins and other birds in the Midwest, as well as throughout the northeast United States.

Pesticides continue to be a serious problem in the world today. The World Health Organization today reports there are 26 million pesticide poisonings including 220,000 deaths per year in the world [2]. Although the United States has some of the best regulations to protect humans and the environment against the hazards of pesticides, the Environmental Protection Agency reports 300,000 non-fatal human pesticide poisonings [3].

## What was the impact of publishing these findings?

Following the evidence of the dangers of pesticide spraying, several investigators in the United States and Canada were concerned with the overuse of pesticides. In particular there was concern over the heavy pesticide use in basic crops such as corn, soybeans, and cotton.

Several investigators and myself supported by the Rodale Institute designed a research project in Pennsylvania to compare the heavy use of pesticides as recommended by Pennsylvania State, with the production of corn and soybeans produced without any pesticides. Both corn and soybean treatments were grown in rotation. The field experiment was run for 22 years and the results were published in the Journal of BioScience [4]. There was no difference in the corn and soybean yields after 22 years of production in the two treatments. Thus, one has to ask why are corn and soybeans heavily treated with pesticides today when they can be produced without any pesticides? The insecticide and herbicide treatments for corn grain costs about $70 per year and about $190 for soybean treatments per hectare.

## What has been the legacy of the book?

After Rachel Carson’s book was published, a few nations implemented programs to reduce pesticide use. For example, Sweden over a 10 year period has been able to reduce pesticide use in the nation by 68% and concurrently reduced public health impacts 77% [5]. In addition, Indonesia was able to reduce pesticide use in rice 65% while increasing rice yields 12% and diminishing production costs [6].

There is a critical need to follow through on Rachel Carson’s recommendations to reduce pesticide use to improve crop production and the profitability of agriculture. This as demonstrated can benefit the economics of crop production while protecting public health and the environment. Sweden and other nations are concerned with the overuse of pesticides and are making efforts to reduce their use in agriculture and to protect their environments and public health.

Looking ahead to the coming decades with the human population rapidly expanding, the need for food and other resources will continue to grow. The need for pest control will also grow in time. It is my view that pesticide use worldwide could be reduced 50% and still achieve effective pest control [7].

## Competing interests

David Pimentel is an Editorial Advisor for *BMC Ecology.*

## Authors’ contributions

DP wrote and approved the final text.
